# Retrieval-Based Evaluation of Cell Painting Feature Spaces Reveals Differences in the Preservation of Biologically Meaningful Phenotypic Similarity

**DOI:** 10.3390/ijms27135727

**Published:** 2026-06-25

**Authors:** Xenia Kuznetsova, Larisa Kuznetsova, Elina Shabunina, Igor Sergeev, Igor Malyshev

**Affiliations:** Laboratory of Cellular Biotechnologies, Russian University of Medicine of the Ministry of Health of the Russian Federation, 4 Dolgorukovskaya St., 127006 Moscow, Russia; xeniakuznetsova@gmail.com (X.K.); eea92@mail.ru (E.S.); serg.igor1997@gmail.com (I.S.); iymalyshev1@gmail.com (I.M.)

**Keywords:** Cell Painting, morphological profiling, feature extraction, mechanism of action (MOA), mean average precision

## Abstract

Cell Painting enables high-dimensional phenotypic profiling of cellular states, but retrieval-based interpretation depends on whether the chosen feature space preserves task-relevant biological relationships. With pretrained Cell Painting feature extractors increasingly available, feature spaces should be qualified before downstream biological retrieval. Here, we developed a task-aware workflow for evaluating Cell Painting feature spaces on a curated U2OS JUMP-MOA reference plate. Three pretrained models, CellPaintSSL, OpenPhenom, and uniDINO, were applied in a zero-shot setting to the same image set, and the resulting profiles were analyzed using a copairs-based mean average precision (mAP) retrieval framework. We assessed compound-induced activity relative to dimethyl sulfoxide (DMSO) controls, same-compound profile resolution among active perturbations, and mechanism-of-action (MOA) annotation recovery at the compound-profile level. All three feature spaces showed strong prerequisite performance, with a large proportion of compounds passing both activity and distinctiveness criteria. However, MOA annotation recovery was partial and model-dependent. Although the overall number of recovered MOA annotations was similar across feature spaces, the specific MOA annotations recovered by each model differed. These results show that prerequisite profile quality does not guarantee recovery of the biological relationship being tested, such as shared MOA annotation, and support task-aware qualification of feature spaces before downstream interpretation.

## 1. Introduction

Among image-based profiling approaches, Cell Painting has emerged as a widely used platform for high-dimensional morphological characterization of cellular states, with applications in cell biology, functional genomics, drug discovery, bioactivity prediction, toxicology, and related areas [1,2,3,4,5,6]. By converting multiplexed fluorescence microscopy images into quantitative representations, it has transformed microscopy from a primarily descriptive method into a source of high-dimensional phenotypic data, enabling cellular states to be described as integrated morphological phenotypes.

A key conceptual foundation for interpreting such data is the guilt-by-association principle, which states that perturbations producing similar morphological profiles are likely to affect shared biological targets, signaling pathways, or cellular states [7,8]. Accordingly, similarity between profiles induced by small molecules or genetic perturbations can be interpreted as evidence of shared biological processes or functionally related molecular targets [8]. In this context, the cell can be viewed as an integrative sensor of molecular perturbations, translating complex intracellular events into observable phenotypic changes.

From this perspective, phenotypic profiling naturally lends itself to retrieval-based evaluation [9]. In such analyses, a perturbation profile is used as a query against a reference set of profiles annotated with known mechanisms of action, genetic perturbations, or other biological labels. The premise of this approach is that, in a biologically meaningful feature space, profiles linked by shared biology tend to appear near the top of the ranking. Although retrieval performance is also affected by the similarity metric used, it depends far more fundamentally on the properties of the feature space itself. This, in turn, makes it essential to assess whether the chosen feature space is appropriate for the biological domain of interest, that is, whether it preserves relevant biological relationships while suppressing technical noise.

This need has brought increased attention to retrieval-aware strategies for feature-space evaluation. One of the most conceptually coherent approaches in this area is the mean average precision (mAP)-based framework proposed by Kalinin et al. [9]. In this framework, different aspects of profile quality and biological similarity can be formulated as retrieval problems, with mAP serving as a unified quantitative measure of retrieval performance. In the present study, this logic was applied to three complementary scenarios: compound-induced activity relative to dimethyl sulfoxide (DMSO) controls, same-compound profile distinctiveness among active perturbations, and recovery of shared mechanism-of-action (MOA) annotations at the compound-profile level. In practice, this analysis can be implemented using copairs, a library designed for efficient computation of mAP and related statistics in large-scale profiling datasets [9,10].

Historically, feature extraction in Cell Painting relied on expert-designed morphometric descriptors generated by classical image-analysis pipelines, most notably CellProfiler [11]. In recent years, however, the field has undergone a marked shift toward deep learning models capable of learning feature representations directly from microscopy images. This transition has substantially expanded the analytical potential of phenotypic profiling, as deep neural networks can capture complex nonlinear patterns that often remain inaccessible to handcrafted morphometric descriptors [12,13,14,15,16,17].

A broad range of open deep learning models for biological feature extraction from microscopy images is now available. These models differ substantially in both architecture and training regime, ranging from specialized encoders developed specifically for the Cell Painting assay, such as CellPaintSSL, to assay-independent generalist models, such as uniDINO [18,19]. The availability of such pretrained models has created a new paradigm in phenotypic profiling in which feature spaces can now be transferred in a zero-shot manner across independent datasets and diverse biological applications. This shift introduces a critical challenge of choosing among available pretrained models for independent use in a specific biological profiling context, especially under experimental conditions that may differ substantially from the model’s original development and evaluation domain. Consequently, it becomes increasingly important to develop rapid, reproducible, and conceptually grounded strategies for assessing whether a given feature space is appropriate for a specific biological profiling context [20]. Such assessment is non-trivial, as it requires tools that enable feature-space suitability to be evaluated in a reproducible, statistically grounded, and conceptually coherent manner rather than on the basis of ad hoc criteria.

In the present study, we develop and demonstrate a workflow for feature-space qualification at the pilot stage of assay development. Specifically, we repurpose Kalinin’s versatile metadata-driven retrieval framework [9] as a diagnostic readout for comparing publicly available pretrained deep models under specific experimental conditions. An important practical advantage of this framework is that it allows the investigator to dynamically alter matching rules based on plate metadata, making it possible to evaluate multiple aspects of model performance within a single analytical scheme.

As a practical annotated benchmark, we used Cell Painting images acquired from U2OS cells on a curated Joint Undertaking for Morphological Profiling (JUMP) MOA reference plate, hereafter referred to as the JUMP-MOA reference plate [21,22]. Within this benchmark, we simulated the task of retrieving compounds with predefined MOA annotations to determine how model choice may depend on the predefined MOAs of interest. Three publicly available pretrained deep learning models—CellPaintSSL, OpenPhenom [23], and uniDINO—were applied in zero-shot mode, with preprocessing and postprocessing performed in accordance with their respective developer recommendations.

Importantly, the aim of the current study was neither to rank neural network architectures in general nor to establish the JUMP-MOA plate as a universal reference for MOA recovery. Rather, the single U2OS JUMP-MOA reference plate was used as a defined proof-of-concept setting to test whether different feature spaces recovered the same or different annotated MOA relationships. We first assessed prerequisite profile quality through activity and distinctiveness scenarios, and then tested MOA annotation recovery directly using a retrieval-based analytical framework implemented in the copairs library [9,10]. This design allowed us to separate prerequisite profile quality from MOA-level retrieval performance and to determine whether different feature spaces supported the same or different predefined MOA relationships.

## 2. Results

### 2.1. Activity

We first evaluated compound activity as the first prerequisite layer of feature-space qualification, defined as the ability of replicate compound-treated wells to retrieve one another against DMSO vehicle-controls. In this scenario, each compound-treated well profile served as a query; wells treated with the same compound were considered positives, whereas DMSO wells were considered negatives. This analysis therefore measures whether a compound induces a reproducible phenotypic profile distinguishable from negative controls.

**Figure 1:**
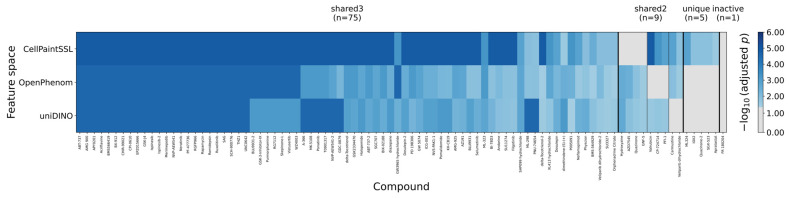
Compound-level activity across feature spaces. The heatmap shows compound-level activity significance for CellPaintSSL, OpenPhenom, and uniDINO. In this scenario, compound-treated well profiles served as queries, replicate wells of the same compound were considered positives, and dimethyl sulfoxide (DMSO) wells were considered negatives. Color represents −log10 of the adjusted *p*-value, while gray cells indicate non-significant compound–feature-space pairs. Compounds are ordered by their activity classification pattern across feature spaces: shared3 denotes compounds significant in all three feature spaces, shared2 denotes compounds significant in exactly two feature spaces, unique denotes compounds significant in only one feature space, and inactive denotes compounds not classified as activity-positive in any feature space. Within each block, compounds are ordered by the summed −log10 adjusted *p*-value across the three feature spaces, from higher to lower values.

Across the full matched set of 90 compounds, the three feature spaces produced similar binary activity classifications. Compounds were classified as activity-positive if their compound-level mAP score was significant after multiple-testing correction in the activity scenario (Benjamini–Hochberg adjusted *p* < 0.05). CellPaintSSL classified 85 of 90 compounds as activity-positive, OpenPhenom classified 81 of 90, and uniDINO classified 82 of 90 (Table 1). At the compound level, these activity classifications were partitioned into four groups: 75 compounds were classified as active in all three feature spaces, 9 in exactly two feature spaces, 5 in only one feature space, and 1 compound was not classified as active in any feature space (Figure 1).

**Figure 2:**
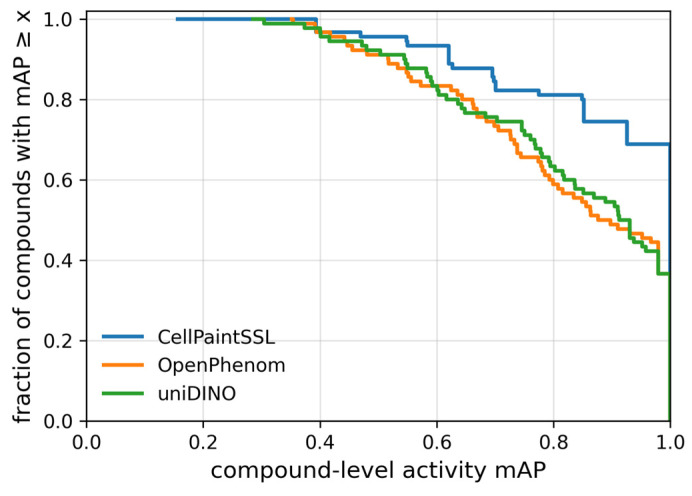
Distribution of compound-level activity retrieval across feature spaces. Reverse empirical cumulative distributions of compound-level mAP values in the activity scenario are shown. The *x*-axis shows compound-level mAP, and the *y*-axis shows the fraction of compounds with mAP greater than or equal to that value across the full matched set of 90 compounds. Each curve corresponds to one feature space. Curves shifted upward and to the right indicate stronger activity retrieval across a larger fraction of compounds. Pairwise Wilcoxon signed-rank tests showed higher activity mAP for CellPaintSSL than for OpenPhenom and uniDINO (adjusted *p* < 0.0001 for both comparisons), whereas OpenPhenom and uniDINO did not differ significantly.

Compound-level activity retrieval was high across all feature spaces but differed in magnitude between them (Figure 2, Table 1). As a performance metric, mAP is bounded between 0 and 1, and in the activity scenario many compounds reached values close to the upper bound. Shapiro–Wilk tests did not support the normality of either the model-specific compound-level mAP distributions or the paired between-model differences. Therefore, matched compound-level mAP values were compared across feature spaces using rank-based paired tests. Across all 90 compounds, the Friedman test showed a significant overall difference between feature spaces (*p* < 0.0001). In pairwise Wilcoxon signed-rank tests with Holm correction, CellPaintSSL showed higher compound-level activity mAP than both OpenPhenom (adjusted *p* < 0.0001) and uniDINO (adjusted *p* < 0.0001), whereas OpenPhenom and uniDINO did not differ significantly.

Together, these results indicate that similar binary activity classifications do not imply equivalent retrieval quality. Although all three feature spaces classified broadly similar numbers of compounds as activity-positive on this curated MOA reference plate, CellPaintSSL provides stronger compound-level activity retrieval across the full matched compound set.

### 2.2. Distinctiveness

We next evaluated compound-level distinctiveness, defined as the ability of same-compound replicate wells to retrieve one another among wells treated with other compounds. In this scenario, each compound-treated well profile served as a query, same-compound replicate wells were considered positives, and wells treated with different compounds were considered negatives. DMSO wells were excluded from this analysis. As the second prerequisite layer, distinctiveness asks whether compound-induced profiles that are detectable relative to DMSO also remain compound-specific among other perturbation profiles, independently of shared MOA annotation.

**Figure 3:**
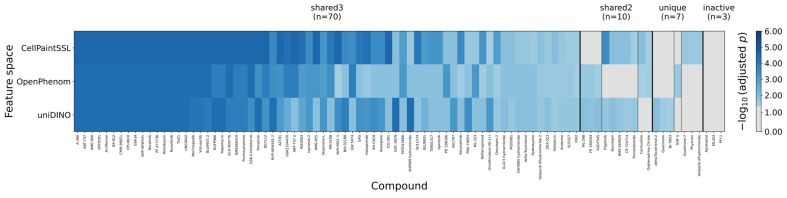
Compound-level distinctiveness across feature spaces. The heatmap shows compound-level distinctiveness significance for CellPaintSSL, OpenPhenom, and uniDINO. In this scenario, compound-treated well profiles served as queries, replicate wells of the same compound were considered positives, and wells treated with different compounds were considered negatives. Color represents −log10 of the adjusted *p*-value, while gray cells indicate non-significant compound–feature-space pairs. Compounds are ordered by their distinctiveness classification pattern across feature spaces: shared3 denotes compounds significant in all three feature spaces, shared2 denotes compounds significant in exactly two feature spaces, unique denotes compounds significant in only one feature space, and inactive denotes compounds not classified as distinctiveness-positive in any feature space. Within each block, compounds are ordered by the summed −log10 adjusted *p*-value across the three feature spaces, from higher to lower values.

Across the full matched set of 90 compounds, most compounds were classified as distinctiveness-positive in each feature space. Compounds were considered distinctiveness-positive if their compound-level mAP score was significant after multiple-testing correction in the distinctiveness scenario (Benjamini–Hochberg adjusted *p* < 0.05). CellPaintSSL classified 80 compounds as distinctiveness-positive, OpenPhenom classified 76, and uniDINO classified 81 (Table 2). These distinctiveness classifications were partitioned into four groups: 70 compounds were significant in all three feature spaces, 10 in exactly two feature spaces, 7 in only one feature space, and 3 compounds were not significant in any feature space (Figure 3).

**Figure 4:**
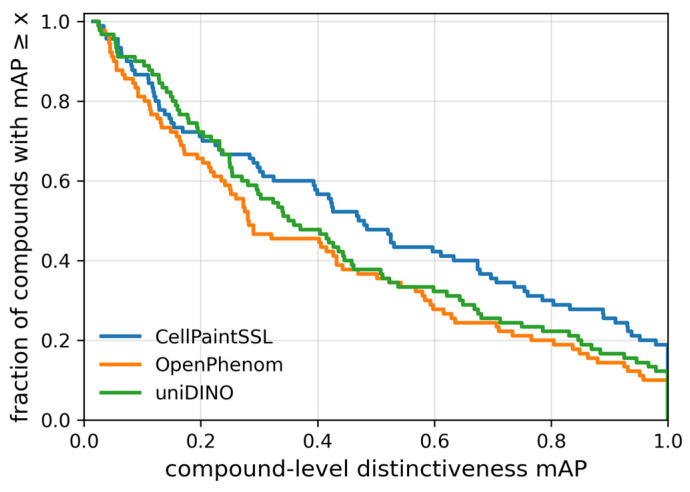
Distribution of compound-level distinctiveness retrieval across feature spaces. Reverse empirical cumulative distributions of compound-level mAP values in the distinctiveness scenario are shown. The *x*-axis shows compound-level mAP, and the *y*-axis shows the fraction of compounds with mAP greater than or equal to that value across the full matched set of 90 compounds. Each curve corresponds to one feature space. Curves shifted upward and to the right indicate stronger distinctiveness retrieval across a larger fraction of compounds. Pairwise Wilcoxon signed-rank tests showed higher distinctiveness mAP for CellPaintSSL than for OpenPhenom (adjusted *p* < 0.0001) and uniDINO (adjusted *p* < 0.01), and higher distinctiveness mAP for uniDINO than for OpenPhenom (adjusted *p* < 0.05).

Compound-level distinctiveness retrieval differed substantially across feature spaces (Figure 4, Table 2). Because mAP is bounded between 0 and 1, distributional assumptions were assessed before feature-space comparison. Shapiro–Wilk tests did not support normality of either the compound-level mAP distributions or the paired between-feature-space differences; therefore, rank-based paired tests were used for formal comparison. Across all 90 compounds, the Friedman test showed a significant overall difference between feature spaces (*p* < 0.0001). In pairwise Wilcoxon signed-rank tests with Holm correction, CellPaintSSL showed higher compound-level distinctiveness mAP than both OpenPhenom (adjusted *p* < 0.0001) and uniDINO (adjusted *p* < 0.01). uniDINO also showed higher distinctiveness mAP than OpenPhenom (adjusted *p* < 0.05).

Together, these results indicate that similar numbers of compounds classified as distinctiveness-positive do not imply equivalent retrieval quality. Although all three feature spaces preserved compound-level identity for many perturbations, CellPaintSSL showed the strongest distinctiveness retrieval overall, followed by uniDINO and then OpenPhenom.

The activity and distinctiveness analyses therefore define complementary prerequisite layers: activity tests whether same-compound replicate profiles are distinguishable from DMSO controls, whereas distinctiveness tests whether they remain compound-specific among other perturbation profiles.

### 2.3. Joint Activity–Distinctiveness Qualification Defines a Model-Dependent Qualified Region

We next combined activity and distinctiveness to define the qualified region of each feature space. Compounds were considered qualified when they were both activity-positive and distinctiveness-positive, corresponding to profiles that were detectable relative to DMSO controls and retained compound-specific identity among perturbation profiles. Distinctiveness-positive classifications almost always overlapped with activity-positive classifications: 79 of 80 distinctiveness-positive compounds in CellPaintSSL, 72 of 76 in OpenPhenom, and 78 of 81 in uniDINO were also activity-positive. The remaining distinctiveness-positive but activity-negative compounds were rare, suggesting that some reproducible compound-specific profiles may nevertheless remain only weakly separated from DMSO controls. Activity-positive but distinctiveness-negative compounds reflected a complementary situation, in which profiles were detectably separated from DMSO controls but did not form sufficiently reproducible compound-specific neighborhoods relative to other perturbation profiles. Overall, the qualified high-activity/high-distinctiveness region contained 79 of 90 compounds for CellPaintSSL, 72 of 90 for OpenPhenom, and 78 of 90 for uniDINO, corresponding to 87.8%, 80.0%, and 86.7% of the matched compound set, respectively (Table 3).

**Figure 5:**
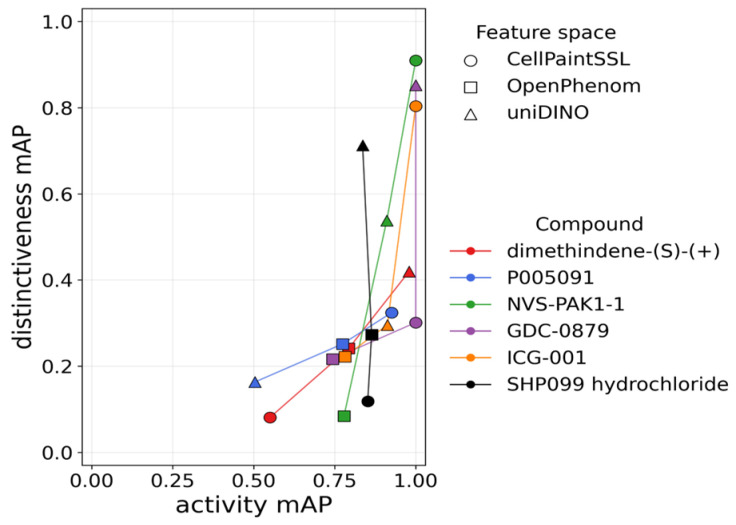
Feature-space-dependent positioning of qualified compounds in activity–distinctiveness space. The scatter plot shows compound-level activity mAP and distinctiveness mAP for selected compounds across CellPaintSSL, OpenPhenom, and uniDINO. Each compound is shown in a distinct color; marker shape denotes the feature space, and points corresponding to the same compound across feature spaces are connected by a thin line. Compounds were selected from the high-activity/high-distinctiveness region based on the largest between-feature-space differences in activity mAP, distinctiveness mAP, or combined two-dimensional displacement. The examples illustrate that compounds passing both prerequisite thresholds can nevertheless differ substantially in retrieval strength between feature spaces. Thus, joint qualification defines an interpretable region for downstream analysis, but it does not imply that individual compound profiles are equivalent across representations.

Although most compounds passed joint qualification on this curated MOA plate, selected qualified compounds occupied widely separated positions in activity–distinctiveness space depending on the feature extractor (Figure 5). These examples show that passing both thresholds does not imply equivalent retrieval strength. The same compound may be strongly activity-positive in all feature spaces but differ substantially in compound-specific distinctiveness, in some cases (e.g., NVS-PAK1-1) ranging from near-threshold values to almost maximal mAP (Figure 5), or may show shifts in both activity and distinctiveness. Thus, joint qualification provides a compact summary of the interpretable phenotypic region, while also revealing that the retrieval reliability of individual perturbation profiles remains feature-space-dependent.

### 2.4. MOA Annotation Recovery as a Downstream Task

After qualifying phenotypic activity and compound-specific distinctiveness, we evaluated whether the feature spaces supported retrieval of compounds sharing MOA annotations. MOA annotation recovery was treated as a downstream retrieval task. Compound-level profiles served as queries; compounds with the same MOA annotation were considered positives, and compounds with different MOA annotations were considered negatives. The analysis was restricted to 86 compounds belonging to MOA classes represented by at least two compounds, corresponding to 43 evaluable MOA annotations. We summarized annotation recovery at two levels: first, as the number of individual compound queries that significantly retrieved compounds with the same MOA annotation, and second, as the number of MOA classes that showed significant recovery after aggregating the mAP values of compound queries sharing the same MOA annotation.

Across all three feature spaces, MOA annotation recovery was partial (Table 4). At the compound-query level, CellPaintSSL and OpenPhenom each identified 17 annotation-positive compound queries out of 86 evaluable compounds, whereas uniDINO identified 15 of 86. At the aggregated MOA level, 9 of 43 evaluable MOA classes were nominally significant in CellPaintSSL and OpenPhenom, corresponding to 20.9% of evaluable MOA classes, whereas 8 of 43 were nominally significant in uniDINO, corresponding to 18.6%. Here, nominal significance refers to unadjusted *p* < 0.05 at the MOA-class level. After multiple-testing correction using the Benjamini–Hochberg procedure, 4 MOA classes remained significant in each feature space.

Most nominally recovered MOA classes were supported by reciprocal compound-level retrieval between paired compounds sharing the same annotation. In CellPaintSSL, 8 of 9 nominally recovered MOA classes had both paired compounds classified as annotation-positive, with the RAF inhibitor supported by only one compound query. In OpenPhenom, 8 of 9 nominally recovered MOA classes were likewise supported by both paired compounds, with IGF-1 inhibitor supported by only one compound query. In uniDINO, 6 of 8 nominally recovered MOA classes were supported by both paired compounds, whereas IGF-1 inhibitor and mTOR inhibitor were supported by only one compound query. Thus, the number of annotation-positive compound queries was similar across feature spaces, but the composition and reciprocity of recovered MOA classes differed between feature spaces.

**Figure 6:**
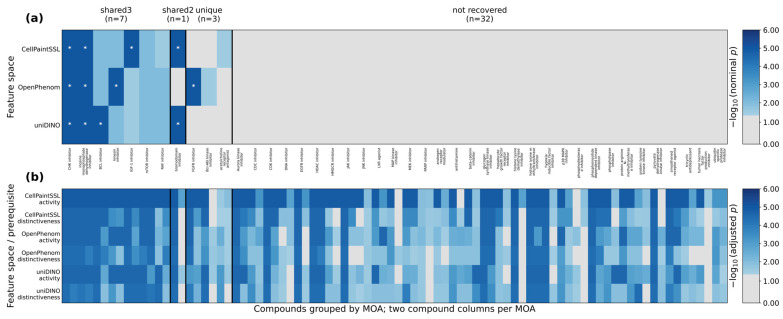
Integrated view of prerequisite qualification and MOA annotation recovery. (**a**) MOA-level annotation recovery across CellPaintSSL, OpenPhenom, and uniDINO. Columns represent evaluable MOA classes, and rows represent feature spaces. Color indicates −log10 of the nominal MOA-level *p*-value; gray cells indicate non-significant results at the nominal threshold. White asterisks mark MOA annotations that remained significant after multiple-testing correction. MOA classes are ordered by their annotation-recovery pattern across feature spaces: shared3 denotes MOA classes recovered at the nominal threshold in all three feature spaces, shared2 denotes classes recovered in exactly two feature spaces, unique denotes classes recovered in only one feature space, and not recovered denotes classes not recovered at the nominal threshold in any feature space. Within each block, MOA classes are ordered by the summed −log10 nominal *p*-value across the three feature spaces. (**b**) Compound-level prerequisite qualification landscape shown in the same MOA order as in panel (**a**). Columns represent individual compounds grouped by MOA class. For each feature space, two rows show compound-level activity and distinctiveness significance, respectively. Color indicates −log10 of the adjusted *p*-value for significant results after multiple-testing correction; gray cells indicate non-significant results. For each MOA class, the two corresponding compounds are displayed as adjacent columns and ordered by decreasing total prerequisite signal across the activity and distinctiveness analyses. Vertical black lines indicate the same MOA-recovery blocks as in panel (**a**).

The recovered MOA annotations overlapped only partially across feature spaces (Figure 6). At the nominal MOA level, some annotations were recovered across multiple feature spaces, whereas others were feature-space-specific, indicating that same-MOA organization depended on the feature representation. After multiple-testing correction, a smaller subset of MOA classes remained significant. CHK inhibitor and inosine monophosphate dehydrogenase inhibitor were significant in all three feature spaces; bromodomain inhibitor was significant in CellPaintSSL and uniDINO; IGF-1 inhibitor was significant only in CellPaintSSL; FGFR inhibitor and kinesin inhibitor only in OpenPhenom; and BCL inhibitor only in uniDINO. These results indicate that MOA recovery was not simply a property of the compound panel itself, but depended on the feature space in which perturbation profiles were represented.

Together, these results show that MOA annotation recovery was partial and feature-space-dependent on the curated MOA reference plate. Across all three feature spaces combined, 49 compound queries were annotation-positive; 47 of these were located in the high-activity/high-distinctiveness qualified region, whereas only 2 were outside this region (Fisher’s exact test, *p* = 0.0061). Notably, the two annotation-positive queries outside the qualified region were not activity-negative profiles. Both were activity-positive PFI-1 profiles that failed only the distinctiveness criterion. The integrated prerequisite–annotation map showed that prerequisite qualification increased the likelihood of MOA annotation recovery but did not determine it. Strong activity and distinctiveness identified profiles for which annotation search could be meaningfully interpreted, yet many such profiles still did not recover a shared MOA label. This indicates that even among qualified perturbation profiles, organization by curated MOA annotation remained incomplete and feature-space-dependent.

## 3. Discussion

The interpretation of Cell Painting profiles is grounded in the idea that morphological similarity can reflect shared biology, and perturbations that produce similar morphological phenotypes may act through related targets, pathways, or cellular states [7,8]. Within this context, a morphological profile can be viewed as an integrated readout of perturbation effects, and phenotypic profiling can be naturally formulated as a retrieval task, in which a query profile is compared with a reference set to identify perturbations with a desired biological activity, infer potential mechanisms of action, or relate cellular states through shared phenotypic patterns [9].

Any retrieval task requires a reference set against which a query profile can be compared. The most ambitious implementation of this idea is atlas-scale phenomic retrieval, exemplified by Recursion’s RxRx3 resource and its compact RxRx3-core release, which provide large-scale image-based maps of genetic and chemical perturbations together with benchmark tasks for perturbation signal and biological relationship retrieval [20,24]. These resources illustrate the power of a large, internally standardized phenomic reference space even when the relevant biological reference is not specified in advance and must instead be inferred from the query’s nearest neighbors in the map. However, the existence of such an atlas does not by itself make annotation of independently generated profiles straightforward. The central difficulty is that an external query profile must first be shown to be biologically interpretable within the atlas system. Its morphology should reflect a genuine perturbation-induced response, and that response must be represented in a way that makes appropriate annotated profiles retrievable in the reference map [9,20]. This challenge motivates ongoing work on approaches that aim to mitigate technical variation and improve profile comparability, including batch-correction methods for image-based profiling and confounder-aware representation or foundation modeling, among other related strategies [14,25,26,27,28]. Nevertheless, a simple, universal workflow in which an independent user can generate a new profile and directly infer its biology from an external atlas remains challenging.

For most research laboratories, a more realistic profiling setting is therefore reference-defined retrieval, in which the biological objective is specified in advance and a compact, purpose-built reference panel can be generated in the same experimental and analytical context. Such a design makes it possible to compare publicly available feature extractors directly and ask which of them is sensitive to the intended biological retrieval relationship. Examples of reference-defined retrieval include searches for perturbations that induce a predefined phenotypic change. The reference may be the phenotype induced by a positive-control compound, a desired transition between cellular states, such as from a disease-like state toward a rescued or healthy-like state, or an undesired anchor phenotype, such as toxicity or stress. Other reference-defined tasks include matching chemical perturbations to a predefined genetic perturbation phenotype or retrieving compounds associated with a specified MOA [2,12,29,30]. In each case, the task involves comparing a query profile with a predefined biological reference, rather than attempting to infer the full biological meaning of the profile from an unrestricted atlas search. The choice of feature space is critical because the representation must make the relevant query–reference relationship retrievable.

The aim of the present study was therefore to develop and demonstrate a workflow for assessing whether a given Cell Painting feature space is suitable for a specific reference-defined retrieval task. Rather than asking which pretrained model is globally best, we asked which of three publicly available Cell Painting feature extractors—CellPaintSSL, OpenPhenom, and uniDINO—produces the feature space most appropriate for retrieving compounds sharing predefined MOA annotations on the JUMP-MOA reference panel. The curated JUMP-MOA plate was used for this purpose because it provides a compact and annotated experimental system for task-aware qualification of Cell Painting feature spaces in a predefined MOA annotation-recovery task [21,22]. It should be noted, however, that the JUMP-MOA reference design is inherently restricted, comprising a single plate, a single cell line, a single concentration, a single time point, and limited compound representation across MOA classes. For the purposes of the present study, these restrictions were acceptable because the goal was not broad cross-condition generalization, but demonstration of divergence in model behavior within a defined reference setting and of a feature-space qualification workflow.

Following the retrieval-based framework proposed by Kalinin et al. [9], we evaluated each feature space using a sequence of mAP-based retrieval scenarios. The first two scenarios were used as prerequisite QC checks for data integrity before MOA-level retrieval [31,32]. The activity scenario assessed whether compound-induced profiles were distinguishable from DMSO controls, reflecting detectable compound-induced phenotypic activity. The distinctiveness scenario assessed whether profiles induced by the same compound retrieved each other among profiles induced by other compounds, reflecting compound-level profile resolution. Collectively, high performance in these prerequisite QC checks was interpreted as evidence that the experimental and computational pipeline preserved detectable and reproducible biological signal, thereby confirming sufficient data integrity for downstream MOA-level retrieval. Recent work has also shown that, for phenotypic profiling tasks influenced by cell health, proliferation, or cytotoxicity, cell or nuclei count can serve as an important additional prerequisite baseline before interpreting high-dimensional profile-based results [33]. The final scenario addressed the reference-defined task itself by testing whether compound-level profiles retrieved other compounds sharing the same MOA annotation. This design separated prerequisite profile qualification from MOA-level biological retrieval.

A first finding of this study is that strong performance of a feature space in prerequisite retrieval scenarios does not necessarily imply sensitivity to a particular predefined biological retrieval task. High activity and distinctiveness scores indicate that the feature space produces high-quality compound-induced profiles—they are detectable relative to control wells and sufficiently resolved among other compound-induced profiles. However, such prerequisite profile quality does not guarantee that the feature space encodes the specific biological relationship required by the task. This task-specific sensitivity must therefore be tested directly.

A second important finding is that task-specific sensitivity was feature-space-dependent despite broadly similar aggregate retrieval outcomes across feature spaces. Comparable aggregate performance should therefore not be taken as evidence of equivalent biological sensitivity.

Indeed, all three feature extractors were applied to the same Cell Painting images from the JUMP-MOA reference plate and evaluated using the same retrieval framework. CellPaintSSL, OpenPhenom, and uniDINO showed strong prerequisite performance, with 79/90, 72/90, and 78/90 compounds located in the high-activity/high-distinctiveness qualified region, respectively, corresponding to 87.8%, 80.0%, and 86.7% of the full matched compound set (Table 3).

When we then examined MOA annotation recovery, the aggregate outcomes were again similar across feature spaces, but much more limited. CellPaintSSL, OpenPhenom, and uniDINO identified 17/86, 17/86, and 15/86 annotation-positive compound queries, respectively. At the MOA-class level, 9/43, 9/43, and 8/43 classes were nominally significant in CellPaintSSL, OpenPhenom, and uniDINO, respectively, corresponding to 20.9%, 20.9%, and 18.6% of evaluable MOA classes, whereas only 4/43 classes per feature space remained significant after multiple-testing correction (Figure 6, Table 4).

These results follow the same general pattern reported in prior Cell Painting benchmarks, where replicate- or activity-based retrieval is consistently stronger than retrieval based on shared biological annotation. In particular, in an assay-optimization study using a JUMP-MOA reference plate, U2OS percent matching was approximately 18–35%, whereas percent replicating was substantially higher, approximately 55–75% [22]. In a cross-instrument Cell Painting benchmark also using a JUMP-MOA reference plate, percent matching was in the 16–26% range, whereas percent replicating was in the 40–70% range across microscope settings [34].

The same gap between activity retrieval and shared-annotation retrieval was also apparent in broader Cell Painting datasets analyzed with the mAP framework by Kalinin et al. [9]. In a matched Cell Painting/nELISA compound dataset, Cell Painting profiles retrieved 72% of compounds in the phenotypic-activity scenario, where compound replicates were retrieved against controls, but only 23% in the same-target-gene consistency scenario, where compounds annotated with the same target gene were retrieved against other compounds. In the cpg0004 small-molecule Cell Painting dataset [35], 34% of compounds were activity-positive; only this active subset was carried forward to the downstream consistency analysis, where 32% of evaluable target-gene groups showed consistent phenotypic similarity. These cross-method and cross-dataset performance metrics are summarized in Table 5, providing a reference point for interpreting our deep learning results in relation to classical CellProfiler-based benchmarks and target-gene consistency analyses.

Thus, our results are consistent with the broader Cell Painting retrieval pattern in which replicate-based profile recovery is relatively strong, whereas retrieval based on shared biological annotation is substantially more selective. Crucially, Table 5 shows that this general pattern is observed across classical feature-based pipelines, different annotation types, and deep self-supervised embeddings. In our study, this distinction was reflected by the high fraction of compounds passing the prerequisite activity/distinctiveness layers and the much smaller fraction of compounds or MOA classes showing significant MOA annotation recovery. We interpret this pattern as evidence that both the cellular protocol and the computational pipelines are functioning within an expected range, thereby serving as a functional QC readout for the workflow.

A plausible explanation for why replicate-based retrieval is stronger than MOA retrieval is that, for the self-supervised deep learning models evaluated here, this pattern may stem from two factors.

Biological dominance of compound identity: distinct compounds sharing an MOA share only a specific biological perturbation, while their remaining phenotypic fingerprint, driven by differences in chemical structure and off-target effects, may vary substantially. This compound-specific signal may overshadow the shared phenotypic features driven by the common mechanism [36]. Consequently, the feature space may preserve identity signatures more strongly than MOA-specific signatures, leading to strong replicate retrieval but weaker retrieval across different compounds with the same MOA.

The self-supervised learning objective: computationally, the models used are explicitly optimized to map augmentations of the same image to nearby points in feature space. Replicate retrieval therefore aligns naturally with this training objective. By contrast, retrieving distinct compounds with the same MOA requires grouping defined by biological annotations, information that is absent during self-supervised training. Consequently, high replicate reproducibility may primarily reflect the model’s ability to recognize identity and does not necessarily guarantee its ability to cluster biologically related but chemically distinct perturbations.

The critical point is that similar aggregate rates of MOA annotation recovery did not mean that the same MOA relationships were recovered by each feature space. At the nominal level, seven MOA annotations were recovered in all three feature spaces, one was recovered in two feature spaces, and three were recovered in only one feature space (Figure 6a). After multiple-testing correction, two MOA annotations, CHK inhibitor and inosine monophosphate dehydrogenase inhibitor, were recovered in all three feature spaces. One MOA-level signal that remained significant after correction, bromodomain inhibitor, was recovered in two feature spaces, CellPaintSSL and uniDINO. The remaining corrected MOA-level signals were feature-space-specific: IGF-1 inhibitor in CellPaintSSL, FGFR inhibitor and kinesin inhibitor in OpenPhenom, and BCL inhibitor in uniDINO (Figure 6a).

Thus, although the number of recovered MOA relationships was nearly the same across feature spaces, the recovered biological relationships were not. The key distinction between feature spaces was, therefore, not the extent of MOA recovery, but the identity of the MOA relationships that each feature space made retrievable.

From a practical decision-making standpoint, these findings imply that model choice should be guided not by aggregate retrieval performance alone, but by recovery of the particular predefined MOAs that are of primary interest for the intended assay (Figure 6). For example, if the downstream application is centered on the recovery of IGF-1 inhibitor-like profiles, the present results would favor CellPaintSSL, whereas assays focused on the recovery of FGFR inhibitor- or kinesin inhibitor-like profiles would favor OpenPhenom under the same benchmark conditions. By contrast, for MOAs such as CHK inhibitor or inosine monophosphate dehydrogenase inhibitor, which were recovered across all three feature spaces, model choice becomes less constrained by the biological target itself. Thus, the practical meaning of the present results is not that one model is universally superior, but that different feature spaces may be preferable for different predefined MOA-retrieval objectives.

Our finding that certain MOA classes are recovered in only one or two of the three evaluated feature spaces (Figure 6) may reflect underlying differences in how these pretrained models process the same Cell Painting images. One possible explanation is that pretrained models view the same microscopic images through distinct representational lenses: some may be more sensitive to high-frequency texture variation within specific cellular compartments, whereas others may place greater weight on global cell shape and overall spatial organization [37,38,39]. Consequently, if the phenotypic signature of a given biological mechanism is encoded predominantly in local organelle granularity, a shape-biased encoder might not retrieve it, and vice versa. Such representational non-equivalence may help explain the only partly overlapping MOA sets recovered here and supports our core conclusion that, if different networks extract different biological signals from the same assay, then a task-aware qualification pipeline becomes necessary to match the feature space to the biological question of interest.

At the same time, retrieval of the target MOAs should not be treated as the sole criterion for model selection in a real pilot workflow. Final model choice should be based on a combination of performance characteristics, including not only recovery of the predefined MOAs of interest, but also the stability of results across independent series, sensitivity to positional effects, the robustness of batch correction, and other assay-level QC considerations where relevant. In practice, the preferred model may therefore be not the one with the highest retrieval signal in isolation, but the one that provides the most reliable overall performance under the intended experimental conditions. The elegance of Kalinin’s framework lies in the fact that all these extensions can be incorporated straightforwardly within the same metadata-driven pairing scheme used in the present manuscript [9].

These findings support a practical workflow for task-aware selection of Cell Painting feature spaces. The first step is not to choose a feature extractor, but to define the intended retrieval task and to construct a compact reference panel that represents the relevant biological relationship. Such a panel should include appropriate controls, replicate structure, and reference perturbations or cellular states that define the query–reference relationship to be tested. Candidate feature extractors can then be applied to the same image set, producing alternative feature spaces for direct comparison.

The evaluation should proceed in layers. Prerequisite scenarios first test whether the profiles are suitable for interpretation. Compound- or condition-induced profiles should show detectable activity relative to controls and sufficient resolution among other active profiles. Only after this qualification step should the downstream biological retrieval task be evaluated directly. In our case, this task was retrieval of compounds sharing MOA annotations. In other settings, the same qualification logic could be applied to phenotype-state conversion tasks, such as disease-to-healthy rescue or other biologically defined state transitions. Indeed, the selected-compound activity–distinctiveness map illustrates why this workflow is not limited to MOA-level retrieval (Figure 5). Even among compounds that passed joint qualification, the same perturbation could occupy different positions in activity–distinctiveness space across feature spaces. For phenotype-state conversion tasks, the relevant diagnostic view may therefore focus not on MOA-class recovery, but on the position and retrieval strength of predefined source- and target-state reference profiles within the qualified region.

The resulting choice of feature space should therefore be task-aware. If several feature spaces pass the prerequisite layers, the criterion for final selection should be whether they make the intended biological relationships retrievable. Overall retrieval strength can guide interpretation, but it should not be treated as a substitute for direct testing of the specific retrieval task. This point is especially important in the current landscape, where pretrained Cell Painting feature extractors are becoming increasingly accessible and are often applied in zero-shot settings; their suitability should be established in the biological context in which they will be used, rather than assumed from general benchmark performance.

## 4. Materials and Methods

**Annotated JUMP-MOA Cell Painting Plate.** As a controlled annotated benchmark, we used Cell Painting images acquired from U2OS cells on a JUMP-MOA plate (SPECS, Zoetermeer, The Netherlands), developed by the JUMP Cell Painting Consortium [21]. The plate contained 90 compounds spanning 47 MOA classes, with four replicate wells per compound and 24 DMSO wells used as negative controls. MOA annotations were obtained from the Broad Drug Repurposing Hub [40]. At the class level, 43 MOA classes were represented by two compounds and 4 MOA classes by a single compound. This design provided a compact, biologically annotated experimental system for comparative retrieval-based evaluation of feature spaces.

**Cell Culture and Compound Treatment.** U2OS cells (ATCC, Manassas, VA, USA) were cultured in DMEM (PanEco, Gorki Leninskiye, Russia) supplemented with 10% fetal bovine serum (Gibco, Waltham, MA, USA) and 1% penicillin-streptomycin (PanEco, Gorki Leninskiye, Russia) at 37 °C in a humidified atmosphere containing 5% CO_2_. Cells were maintained in T75 flasks (Nest, Wuxi, China) and harvested at approximately 80% confluency. They were then seeded into 384-well PhenoPlates (Revvity, Waltham, MA, USA) at a density of 4000 cells per well in 30 µL of culture medium.

After 24 h, 10 µL of either DMSO vehicle (MedChemExpress, Shanghai, China) or compound solution was added to each well. Compounds were applied at a final concentration of 3 µM, and the final DMSO concentration did not exceed 0.15%. Cells were incubated with compounds for 24 h prior to staining and imaging.

**Cell Painting Staining and Image Acquisition.** Cell staining was performed according to the optimized Cell Painting protocol [22] using the PhenoVue Cell Painting JUMP Kit (Revvity, Waltham, MA, USA). Live U2OS cells were first incubated with MitoTracker Deep Red by adding 20 µL of a 1.5 µM staining solution to each well containing 40 µL of culture medium, followed by incubation for 30 min at 37 °C. Cells were then fixed by adding 20 µL of 16% paraformaldehyde (Electron Microscopy Sciences, Hatfield, PA, USA) and incubating for 20 min at room temperature, followed by four washes with Hanks’ balanced salt solution (HBSS) (80 µL per well, with final aspiration).

Cells were subsequently incubated for 30 min at room temperature in the dark with 20 µL per well of staining and permeabilization solution prepared immediately before use. The solution contained Hoechst 33342 (1 µg/mL), Concanavalin A conjugated to Alexa Fluor 488 (5 µg/mL), SYTO 14 nucleic acid stain (6 µM), wheat germ agglutinin conjugated to Alexa Fluor 555 (1.5 µg/mL), and phalloidin conjugated to Alexa Fluor 568 (8.25 nM). The solution was prepared in PhenoVue Dye Diluent A supplemented with Triton X-100 (0.1% *v*/*v*; Sigma-Aldrich, Burlington, MA, USA). After staining, cells were washed four times with HBSS (80 µL per well, without final aspiration), and the plate was then sealed and stored at 4 °C until imaging.

Images were acquired using an Operetta high-content imaging system (Revvity, Waltham, MA, USA) with a 20× water-immersion objective. Nine fields of view were collected per well. Monochrome TIFF images were acquired in six channels, including five fluorescence channels and one brightfield channel, at a resolution of 1024 × 1360 pixels. Details of the fluorescence channel settings are summarized in Table 6. Phalloidin/Alexa Fluor 568 and wheat germ agglutinin/Alexa Fluor 555 signals were imaged in the same fluorescence channel.

**Deep Feature Extraction Models.** Three deep learning models were evaluated in this study: CellPaintSSL [18,41], OpenPhenom [23,42], and uniDINO [19,43]. These models were selected to represent different strategies for deriving feature representations from Cell Painting images. The comparison performed in this study should be understood as a comparison of complete feature-extraction pipelines, including model inference and the corresponding preprocessing and postprocessing procedures, rather than as a comparison attributable solely to the model architecture. CellPaintSSL is a specialized encoder developed specifically for the five-channel Cell Painting assay, OpenPhenom is a model optimized for Cell Painting images but featuring a channel-agnostic design, and uniDINO is an assay-independent generalist model.

For all three models, feature extraction was performed in a zero-shot setting. Pretrained weights released by the developers were used directly, with no additional model training, weight adaptation, or fine-tuning on the study data. Inference was performed to obtain image-derived embedding vectors for downstream retrieval-based analysis.

**Model-Specific Image Preprocessing and Feature Extraction.** For all three models, preprocessing followed the workflows recommended by the respective model developers.

For CellPaintSSL, input data for inference were prepared by linking images to plate map annotations and converting single-channel TIFF images into five-channel TIFF stacks using an explicit channel map corresponding to the Cell Painting fluorescence panel. For each channel, intensities were clipped at the 0.1 and 99.9 percentiles and rescaled prior to stack assembly. An Otsu threshold was additionally computed for the DNA channel and stored in the TIFF metadata. This threshold was used to sample non-empty crops based on a minimum foreground area of 1% in the DNA channel [18,41].

For OpenPhenom, a per-plate field-of-view manifest was first constructed from Operetta TIFF files and the plate map. The selected channels were then converted from uint16 TIFF images into 8-bit model inputs tiled into 256 × 256 crops by clipping intensities at the 0.1 and 99.9 percentiles, followed by linear intensity scaling prior to inference [23,42].

For uniDINO, input data for inference were prepared by constructing a field-of-view manifest from Operetta TIFF files and the plate map. The manifest linked each retained field of view to the corresponding image paths and annotations. Image loading and subsequent model input preparation were handled within the inference workflow itself [19,43].

**Model-Specific Postprocessing and Well-Level Embeddings.** For all three models, postprocessing followed workflows recommended by the model developers.

For CellPaintSSL, raw well-level embeddings were postprocessed using a three-step workflow. First, low-variance features (<1 × 10^−5^) were removed. Dataset-wide sphering was then performed using negative controls as the reference set. Finally, the sphered profiles were normalized at the plate level using median absolute deviation (MAD)-based robust scaling with all wells on the plate as the reference set. The resulting normalized well-level embeddings were used for downstream comparative analysis [18,41].

For OpenPhenom, raw well-level embeddings were postprocessed using a principal component analysis-total variation normalization (PCA-TVN) workflow following the referenced implementation [44]. Embeddings were first normalized relative to control wells, then projected into a control-defined principal component space, and finally aligned to the control covariance structure. The resulting processed embeddings were used for downstream comparative analysis [23,42].

For uniDINO, raw well-level embeddings were first normalized at the plate level and then postprocessed using a PCA-TVN workflow. In this procedure, control-based centering and scaling were applied after plate normalization, principal components were fitted on control wells and used to transform all wells, and the transformed embeddings were subsequently aligned to the control covariance structure. The resulting processed embeddings were used for downstream comparative analysis [19,43].

After model-specific postprocessing, all outputs were reformatted into a unified well-level table structure for downstream comparative analysis. Each row represented a single well-level profile. Shared experimental metadata derived from the same plate map were retained across feature spaces, whereas model-specific technical columns were removed during standardization. Embedding dimensions were additionally stored using a unified feature naming convention.

This harmonized structure enabled the direct application of the same downstream retrieval-based analysis workflow across all compared feature spaces.

**Retrieval-Based Evaluation Framework.** Downstream comparative analysis was performed within a retrieval-based framework using the copairs library [9,10]. In this setting, each well-level profile was treated as a query against a reference set of profiles, and similarity relationships were evaluated according to biologically defined matching criteria. This approach enabled a unified assessment of how well different feature spaces preserved perturbation-related structure in the data.

Retrieval-based evaluation was chosen because the central downstream task in phenotypic profiling is not only profile generation, but also the identification of biologically meaningful neighbors in representation space. Accordingly, feature-space performance was assessed in terms of its ability to retrieve relevant profiles under different biological definitions of relevance.

All analyses were performed on harmonized well-level embedding tables using a common metadata schema and a unified feature representation, allowing direct comparison of retrieval performance across the three feature spaces.

**Retrieval-Based Evaluation Scenarios.** Retrieval performance was evaluated under several complementary scenarios designed to capture different aspects of biological structure in the feature space (Table 7). The scenarios differed in the definition of relevant matches, the evaluation set, and the level at which query-level average precision (AP) values were summarized as mAP.

In all scenarios, query-level AP values were aggregated to mAP at the level indicated in the table. In the activity and distinctiveness scenarios, compounds with significant compound-level mAP after multiple-testing correction were classified as activity-positive and distinctiveness-positive, respectively (Benjamini–Hochberg adjusted *p* < 0.05). The joint high-activity/high-distinctiveness qualified region was then defined within each feature space as the set of compounds classified as both activity-positive and distinctiveness-positive. In the MOA annotation recovery scenario, recovery was summarized both at the compound level and at the MOA-class level. MOA-class-level recovery was assessed by aggregating the mAP values of compound queries sharing the same MOA annotation.

**Retrieval Metrics and Statistical Evaluation.** Profile similarity was quantified using cosine similarity within the copairs-based retrieval framework. Retrieval performance was first evaluated using AP at the query level and then summarized as mAP at the compound or MOA-class level, depending on the scenario. Statistical significance was assessed using null distributions of size 100,000 generated under random ranking for the corresponding query configurations, defined by the numbers of positive and total candidates. For mAP-based analyses, group-level null distributions were obtained by averaging the corresponding query-level null distributions across queries within each group. Multiple-testing correction was performed using the Benjamini–Hochberg procedure, and adjusted *p*-values below 0.05 were considered significant.

For feature-space-level comparisons of retrieval strength, matched compound-level mAP values were compared across CellPaintSSL, OpenPhenom, and uniDINO. Because mAP is bounded between 0 and 1 and many values approached the upper bound, distributional assumptions were assessed using Shapiro–Wilk tests applied to feature-space-specific mAP distributions and paired between-feature-space differences. Because normality was not supported, retrieval strength was compared using non-parametric paired tests. Overall differences across the three feature spaces were assessed using the Friedman test, followed by pairwise Wilcoxon signed-rank tests with Holm correction.

Binary activity, distinctiveness, and annotation classifications were summarized descriptively as counts and percentages. To assess whether annotation-positive compound queries were enriched in the high-activity/high-distinctiveness region, compound–feature-space cases were classified according to joint prerequisite status and annotation status, and enrichment was tested using Fisher’s exact test. For this test, a *p*-value below 0.05 was considered statistically significant.

**Software and Computational Environment.** All downstream analyses were performed using custom Python scripts in Python 3.11.15. Retrieval-based AP/mAP calculations were carried out using copairs v0.5.4. Data processing was performed with pandas v3.0.2 and NumPy v2.4.4, statistical analyses with SciPy v1.17.1, and figures were generated with Matplotlib v3.10.8.

## 5. Conclusions

Our results support task-aware qualification of Cell Painting feature spaces as a prerequisite for retrieval-based phenotypic analysis. A feature space may show strong activity and distinctiveness performance while recovering only a subset of the biological relationships required for a specific retrieval task. Therefore, the choice of feature space should be based on direct testing against a purpose-built reference panel that reflects the intended query–reference relationship. This strategy provides a practical route for selecting among feature spaces generated by pretrained Cell Painting models before screening or downstream biological interpretation.

## Figures and Tables

**Table 1:** Summary of compound-level activity retrieval across the full matched compound set of 90 compounds.

Feature Space	Activity-Positive Compounds, *n* (%)	Median mAP [Q1–Q3] ^1^	Range
CellPaintSSL	85 (94.4)	1.000 [0.852–1.000]	0.156–1.000
OpenPhenom	81 (90.0)	0.870 [0.673–1.000]	0.351–1.000
uniDINO	82 (91.1)	0.912 [0.688–1.000]	0.285–1.000

^1^ mAP, mean average precision; Q1 and Q3 indicate the first and third quartiles, respectively.

**Table 2:** Summary of compound-level distinctiveness retrieval across the full matched set of 90 compounds.

Feature Space	Distinctiveness-Positive Compounds, *n* (%)	Median mAP [Q1–Q3]	Range
CellPaintSSL	80 (88.9)	0.469 [0.147–0.890]	0.016–1.000
OpenPhenom	76 (84.4)	0.281 [0.127–0.626]	0.022–1.000
uniDINO	81 (90.0)	0.345 [0.178–0.679]	0.018–1.000

**Table 3:** Overlap between activity-positive and distinctiveness-positive compounds across feature spaces ^1^.

Feature Space	Activity- Positive	Distinctiveness- Positive	Activity- and Distinctiveness-Positive	Activity-Positive Only	Distinctiveness-Positive Only	None
CellPaintSSL	85	80	79	6	1	4
OpenPhenom	81	76	72	9	4	5
uniDINO	82	81	78	4	3	5

^1^ Values indicate the number of compounds. “None” denotes compounds that were neither activity-positive nor distinctiveness-positive.

**Table 4:** Summary of mechanism-of-action (MOA) annotation recovery across 86 evaluable compounds and 43 evaluable MOA classes.

Feature Space	Annotation-Positive Compound Queries, *n* (%)	Nominally Significant MOA Classes, *n* (%)	MOA Classes Significant After Multiple-Testing Correction, *n* (%)
CellPaintSSL	17 (19.8)	9 (20.9)	4 (9.3)
OpenPhenom	17 (19.8)	9 (20.9)	4 (9.3)
uniDINO	15 (17.4)	8 (18.6)	4 (9.3)

**Table 5:** Comparison of replicate-based and annotation-based retrieval performance with prior Cell Painting benchmarks ^1^.

Study/Feature Extraction Method	Dataset	Replicate-Based Metrics	Annotation-Based Metrics	Key Finding
Cimini et al. [21]/CellProfiler	JUMP-MOA reference plate	percent replicating: ~55–75%	percent matching: ~18–35%	replicate-based recovery was substantially stronger than MOA-based matching
Tromans-Coia et al. [31]/CellProfiler	JUMP-MOA reference plate	percent replicating: ~40–70%	percent matching: ~16–26%	replicate-based recovery was substantially stronger than MOA-based matching
Kalinin et al. [9]/CellProfiler	306-compound Cell Painting dataset	high-activity compounds: 72%	shared target-gene consistency: 23%	activity retrieval was stronger than shared target-gene retrieval
Kalinin et al. [9]/CellProfiler	cpg0004 Cell Painting dataset	high-activity compounds: 34%	shared target-gene consistency: 32% of evaluable target-gene groups in the active subset	activity retrieval was stronger than shared target-gene retrieval
Present study/CellPaintSSL	JUMP-MOA reference plate	high-activity/high-distinctiveness compounds: 87.8%	annotation-positive MOA classes: nominal 20.9%, corrected 9.3%	strong prerequisite retrieval, but substantially weaker MOA annotation recovery
Present study/OpenPhenom	JUMP-MOA reference plate	high-activity/high-distinctiveness compounds: 80.0%	annotation-positive MOA classes: nominal 20.9%, corrected 9.3%	strong prerequisite retrieval, but substantially weaker MOA annotation recovery
Present study/uniDINO	JUMP-MOA reference plate	high-activity/high-distinctiveness compounds: 86.7%	annotation-positive MOA classes: nominal 18.6%, corrected 9.3%	strong prerequisite retrieval, but substantially weaker MOA annotation recovery

^1^ MOA, mechanism of action; JUMP-MOA, Joint Undertaking for Morphological Profiling mechanism-of-action reference plate; cpg0004, the identifier of a Cell Painting Gallery dataset [35] analyzed by Kalinin et al. [9]; percent replicating, retrieval of replicate profiles of the same compound; percent matching, retrieval of different compounds sharing the same annotated MOA; shared target-gene consistency, retrieval of compounds annotated with the same target gene.

**Table 6:** Fluorescent dyes, targeted cellular structures, and imaging channel settings.

Dye	Cellular Structure	Excitation Wavelength, nm/Channel Number	Emission Wavelength, nm
Hoechst 33342	nuclear DNA	405/2	435–480
Concanavalin A/Alexa Fluor 488	endoplasmic reticulum	488/3	500–550
SYTO 14 nucleic acid stain	nucleoli, cytoplasmic RNA	488/4	570–630
Phalloidin/Alexa Fluor 568 ^1^	actin	561/5	570–630
Wheat germ agglutinin/Alexa Fluor 555 ^1^	Golgi apparatus and plasma membrane	561/5	570–630
MitoTracker Deep Red	mitochondria	640/6	650–760

^1^ Phalloidin/Alexa Fluor 568 and wheat germ agglutinin/Alexa Fluor 555 signals were acquired in the same fluorescence channel (channel 5) and therefore share the same imaging settings.

**Table 7:** Retrieval-based evaluation scenarios.

Scenario	Query Unit	Positive Matches	Negative Matches	mAP Aggregation Level	Evaluation Set
Activity	well-level profile	replicate wells of the same compound	DMSO wells	compound level	full matched set of 90 compounds
Distinctiveness	well-level profile	replicate wells of the same compound	wells treated with different compounds	compound level	full matched set of 90 compounds; DMSO wells excluded
MOA annotation recovery	compound-level profile	other compounds with the same MOA	compounds with different MOA	compound level; MOA-class level	86 compounds from MOA classes represented by at least two compounds

## Data Availability

The data supporting the reported results are openly available in the Zenodo repository U2OS JUMP-MOA feature-space qualification dataset at doi:10.5281/zenodo.20027968. The repository contains the postprocessed model-derived feature profiles used as input to the downstream retrieval analyses, harmonized copairs-ready profiles, retrieval-analysis outputs, statistical results tables, figure source data, and custom Python scripts used for profile harmonization, retrieval scenario definition, statistical analysis, and plotting. The CellPaintSSL, OpenPhenom, and uniDINO feature extractors were applied in a zero-shot setting using their public implementations, as described in the Materials and Methods.

## Abbreviations

The following abbreviations are used in this manuscript:

DMSO	Dimethyl sulfoxide
JUMP	Joint Undertaking for Morphological Profiling
HBSS	Hanks’ balanced salt solution
mAP	Mean average precision
MOA	Mechanism-of-action
AP	Average precision
